# Implementation of a Multi-Site Digitally-Enhanced School Clinician Training and ADHD/ODD Intervention Program in Mexico: Randomized Controlled Trial of CLS-R-FUERTE

**DOI:** 10.1007/s41347-023-00367-6

**Published:** 2024-01-09

**Authors:** Lauren M. Haack, Linda J. Pfiffner, Sabrina M. Darrow, Jasmine Lai, Dulce Karely Alcaraz-Beltrán, Jassiel Ulises Martínez-Beltrán, Elva Moreno-Candil, Korinthya Delgado-García, María Fernanda Arriaga-Guerrero, Dulce Maria Ledesma-Saldaña, Maria Elena Urquídez-Valdez, Eva Angelina Araujo

**Affiliations:** 1 Department of Psychiatry and Behavioral Sciences, University of California, San Francisco (UCSF), 675 18Th Street, 94107 San Francisco, CA, USA; 2 Department of Psychology, Universidad Autónoma de Sinaloa (UAS), Ángel Flores s/n Pte. Edificio Central Colonia Centro, Culiacán, Sinaloa, Mexico

**Keywords:** ADHD, ODD, School, Training, Youth, Evidence-based treatments

## Abstract

Childhood conditions of inattention and disruptive behavior, such as Attention-Deficit/Hyperactivity Disorder (ADHD) and Oppositional Defiant Disorder (ODD), are prevalent but undertreated worldwide. One promising solution is harnessing digital technology to enhance school clinician training and ADHD/ODD intervention programs. We conducted a school-clustered randomized controlled trial of CLS-R-FUERTE: a program featuring training/consultation for school clinicians to deliver a six-week intervention comprised of weekly parent and student skills groups, as well as support teachers’ classroom management in the form of a Daily Report Card, all facilitated by electronic program manuals/materials and videoconferencing. A total of *N* = 163 (*n* = 6–8 students/school [ages 6–12] and their parents, teachers, and school clinicians) participated across eight public schools in Sinaloa, Mexico. We randomly assigned four schools to receive CLS-R-FUERTE immediately and four schools to receive school services as usual. We compared groups post-treatment on parent- and teacher-rated ADHD/ODD and impairment. We observed the program implementation in vivo, tracking trainer and school clinician program fidelity, as well as participant attendance and adherence, to evaluate feasibility. We also collected participant ratings of satisfaction and usability on the System Usability Scale to evaluate acceptability. Our CLS-R-FUERTE trial demonstrated high rates of program feasibility and acceptability comparable to prior in-person program trial findings. Students receiving CLS-R-FUERTE showed greater improvement in parent- and teacher-rated ADHD, as well as parent-rated ODD and impairment, compared to students receiving school services as usual. Results support the utility of global digital mental health programs training school clinicians to implement ADHD/ODD interventions, which have the potential to increase evidence-based treatment access and uptake across worldwide contexts.

## Introduction

Childhood conditions of inattention and disruptive behavior, such as Attention-Deficit/Hyperactivity Disorder (ADHD) and Oppositional Defiant Disorder (ODD), impact approximately 7–10% of youth worldwide, respectively (American Psychiatric Association, 2022; Canino et al., 2010; Willcutt, 2012), with estimates suggesting 23–54% of children with ADHD also demonstrate ODD comorbidities (Elia et al., 2008; Jensen et al., 2001; Reale et al., 2017). Fortunately, decades of research support the efficacy and effectiveness of behavioral interventions alone or in combination with medication (DuPaul et al., 2020; Pfiffner & Haack, 2014; Sibley et al., 2023). Behavioral evidence-based treatments (EBTs) target ADHD/ODD symptoms and related impairment via parent training, child skills training, and/or classroom management often in the form of a Daily Report Card (DRC; DuPaul et al., 2020; Pfiffner & Haack, 2014; Sibley et al., 2023).

Unfortunately, only a small minority of youth impacted by ADHD/ODD receive EBTs (Danielson et al., 2018; DuPaul et al., 2020; Vander Stoep & Myers, 2013). High levels of unmet need are perpetuated by limited mental health provider training, contributing to a global shortage of EBTs offered in accessible and trusted settings, such as schools or primary care (DuPaul et al., 2020; Kilbourne et al., 2018). Technology-enabled EBT training programs may offer a solution, particularly for providers of youth in rural or under-resourced areas (Fortuna, 2022; McCarty et al., 2015; Mitrani et al., 2021). In the United States (USA) and Canada, a number of web-based clinician training programs have been successfully developed to promote delivery of empirically-supported mental health services (see Frank et al., 2020; Jackson et al., 2018 for reviews), including a remote web-based training for clinicians delivering in-person ADHD treatments in schools (Pfiffner et al., 2023). Advancements also have been made in the development of global web-based mental health training programs for providers from various backgrounds (Acharya et al., 2017; Bennett‐levy et al., 2012; Fairburn et al., 2017; Heck et al., 2015; Kasparik et al., 2022).

Even when services from trained providers are available, many families may have difficulty engaging in traditional EBTs. Barriers identified in previous research with Spanish-speaking families (a population with persistent health disparities in service utilization for inattention and disruptive behaviors) include limited community knowledge and stigma about ADHD/ODD, lack of insurance coverage or ability to pay out-of-pocket for services, and difficulty securing transportation or time off work (Araujo et al., 2017; Eraldi et al., 2006; Gerdes et al., 2014). Digital mental health interventions offer a promising alternative, given that they can be tailored to fit the needs of under-served communities (Schueller et al., 2019).

The Collaborative Life Skills Remote Program: Familias Unidas Emprediendo Retos y Tareas para el Éxito/Families United in Undertaking Challenges for Success (i.e., CLS-R-FUERTE) is a digitally-enhanced Spanish-language school clinician training and comprehensive ADHD/ODD intervention featuring parent training groups, student skills training groups, and classroom management with a DRC system. CLS-R-FUERTE was designed to address many of the EBT barriers outlined above by not only facilitating school clinician training with technology (i.e., electronic manual/materials and training/consultation via videoconferencing) but also by allowing participants to engage in the intervention remotely via videoconferencing. In addition, because all intervention components are delivered by school clinicians, once trained, they may sustainably offer the digitally-enhanced intervention again and again in their schools at no-cost to a wide array of students, even those without formal inattention and/or disruptive behavior disorder diagnoses. CLS-R-FUERTE was adapted from the in-person CLS-FUERTE program for Mexican school clinicians (Haack et al., 2020) and the CLS-Remote web-based training program for US school clinicians (Pfiffner et al., 2023). Next, the program was pilot tested in three public elementary schools in Sinaloa, Mexico, using implementation science processes grounded in an iterative behavioral intervention design (Czajkowski et al., 2015; Haack et al., in press). The CLS-R-FUERTE pilot evaluation and iterative refinements included consideration of cultural responsivity and safety of procedures and team members involved, as well as the participants’ health literacy and technology proficiency, to ensure attunement of digital strategies to the context (as recommended by Eustis et al., 2023; Mitrani et al., 2021).

Open trial pilot testing demonstrated high rates of CLS-R-FUERTE program feasibility (as measured by school clinician fidelity to the intervention, as well as participant attendance and adherence), acceptability (as measured by participant satisfaction ratings and usability ratings on the System Usability Scale [SUS]; Brooke, 1996), and preliminary effectiveness as a precursor to efficacy testing (Haack et al., in press). Many of the participating students showed reliable change in ADHD/ODD symptom counts and the pre-post effect sizes for ADHD symptom and impairment severity improvement were comparable to trial findings from the in-person Collaborative Life Skills (CLS) school-home programs from which CLS-R-FUERTE was adapted (Haack et al., 2019, 2020; Pfiffner et al., 2016, 2023). For a full description of the CLS-R-FUERTE development, training intervention program description, and open-trial findings, see Haack et al. (in press).

Promising open-trial CLS-R-FUERTE findings are aligned with studies suggesting at least similar (if not superior) results for remote versus in-person clinician training programs and ADHD/ODD interventions (e.g., Dimeff et al., 2009; Khanna & Kendall, 2015; Pfiffner et al., 2023; Xie et al., 2013). That said, without efficacy testing employing a control condition, it is unknown if the CLS-R-FUERTE program produces results in student ADHD/ODD outcomes above and beyond the impact of other factors (for example, student maturation due to the passage of time or employment of traditional school services). Evaluating the CLS-R-FUERTE program in a randomized controlled trial (RCT) is a crucial next step to warrant future implementation and dissemination efforts combatting the widespread impact of ADHD and ODD across cultures.

## Current Study

Our current study aims were to investigate the CLS-R-FUERTE program feasibility, acceptability, and outcomes in an eight-school clustered randomized controlled trial (RCT) comparing students actively receiving the CLS-R-FUERTE program to students receiving school services as usual (SAU) while waiting to receive the CLS-R-FUERTE program. We predicted that the RCT would reveal high rates of CLS-R-FUERTE program fidelity, attendance, adherence, satisfaction, and usability. We also predicted that students randomly assigned to receive CLS-R-FUERTE immediately would improve significantly across ADHD/ODD outcome domains relative to students randomly assigned to SAU.

## Methods

The current study utilized a school-cluster RCT design to examine the impact of treatment assignment (CLS-R-FUERTE or SAU) on student outcomes (i.e., ADHD and ODD symptom severity, as well as overall impairment, as rated by parents and teachers). In schools randomly assigned to the CLS-R-FUERTE condition, we observed the program implementation in vivo, tracking trainer and school clinician program fidelity, as well as participant attendance and adherence. We also collected participant ratings of satisfaction and usability on the System Usability Scale. See below for a more detailed description of the participants, procedures, intervention program, study design, and data analysis plan.

### Participants and Procedures

A total of *N* = 163 (i.e., 6–8 students at each of eight schools totaling *N* = 57 students, as well as their parents (*N* = 57), teachers (*N* = 37), and school clinicians (*N* = 12) participated during the 2022–2023 school year. One parent/caregiver (hereon referred to as “parent”) and one teacher per student were designated as “primary,” meaning they would participate in the program activities (see Table 1 for overview) and complete all measures; each teacher could participate with up to two students in the program. One to two school clinicians per enrolled school who agreed to engage in program activities participated in the current study and completed informed consent procedures approved by the University of California, San Francisco (UCSF) and the Autonomous University of Sinaloa (UAS). In the current study, participating school clinicians were professionals with college or graduate degrees embedded within each school’s “regular education support services unit,” to serve students identified with academic and/or behavioral health needs. See Table 1 for a description of school clinician responsibilities in the CLS-R-FUERTE training and intervention program.

Student recruitment procedures matched that of the in-person CLS-FUERTE trial in Mexico (Haack et al., 2020). Specifically, school personnel identified students with attention and/or behavior concerns in grades 1–5; our team’s clinical research staff completed screening procedures with parents and teachers of identified students who were interested in the program. Parents and teachers of screened students completed informed consent procedures approved by UCSF and UAS Committees on Human Research and also completed ratings of ADHD/ODD and related impairment, including the Child Symptom Inventory (CSI-4; Gadow & Sprafkin, 1997) and the Impairment Rating Scale (IRS; Fabiano et al., 2006) at baseline and post regardless of treatment assignment; they were incentivized with $10 gift cards each time they completed questionnaires, totaling $20. All screening and informed consent activities occurred via secure Zoom videoconferencing and Docusign technology; screening questions were conducted via telephone calls with our clinical research staff, and all study ratings were gathered via secure Qualtrics survey tool technology.

Students were eligible to participate in the current study if they met the following inclusion criteria: (a) at least six CSI-4 inattention symptoms and/or six hyperactive/impulsive symptoms endorsed by the parent or teacher as occurring often or very often, (b) at least one area of IRS functioning rated as ≥ 3 by both parent and teacher, reflecting cross-setting impairment, and (c) a parent and a teacher agreeing to participate; each school could enroll up to eight students for the trial and teachers could have up to two students in the trial. Students taking medication were eligible as long as parents indicated they did not anticipate changes to the medication regimen during the trial period. Exclusion criteria included: significant visual or hearing impairments, severe language delay, psychosis, or pervasive developmental disorder, or needs requiring placement in full-day special classrooms, thus impeding ability for student group engagement. Eligible students provided assent before participating.

**Fig. 1:**
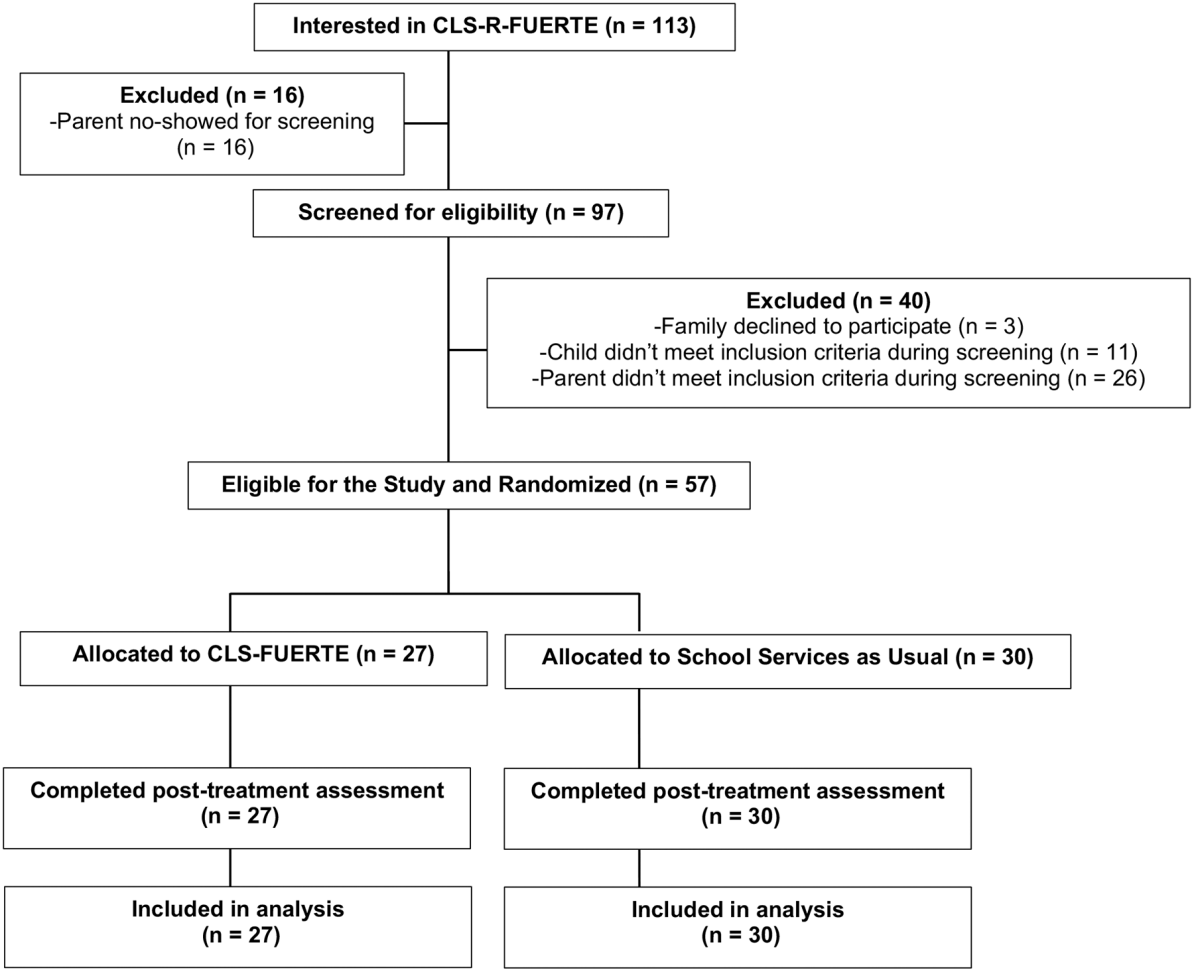
Student participant flow and treatment group randomization

Students were in grades 1–5; they were predominately male (75%) with an average age of 8 years old. Based on parent and teacher baseline ADHD and ODD ratings, 82% demonstrated profiles reflective of ADHD, combined presentation and 49% also demonstrated profiles reflective of ODD presentation. Parents were predominately biological mothers (93%) with varying marital and employment status, as well as levels of educational background. See Table 2 for additional participant demographic information (which was collected on screening interviews) and Fig. 1 for participant flow.

### ADHD/ODD School Clinician Training and Intervention Program: CLS-R-FUERTE

As seen in Table 1, CLS-R-FUERTE is based off of the Collaborative Life Skills (CLS) school clinician training and intervention program designed to improve elementary school student attention and behavior (Pfiffner et al., 2011, 2016), which was subsequently adapted for remote clinician training (i.e., CLS-Remote or CLS-R; Pfiffner et al., 2023) and for Spanish speaking families in the USA (i.e., CLS-Spanish or CLS-S; Haack et al., 2019) and Mexico (i.e., CLS-FUERTE; Haack et al., 2020). In CLS-R-FUERTE, participating school clinicians (see “Participants” section above for description of qualifications) are trained by a clinical research team to support their implementation of a six-week digitally-enhanced ADHD/ODD behavioral intervention incorporating ongoing DRC classroom management by teachers, as well as weekly skill groups for parents and students. School clinicians attend an initial workshop and weekly consultation meetings with their trainer(s) via videoconferencing to learn about inattention/disruptive behavior concerns and underlying ADHD/ODD intervention principles, as well as preview and role-play upcoming intervention components. The intervention component manual and materials are provided via electronic slidedecks, which visually present intervention strategies to participants via infographics and skill demonstration videos; electronic pdf’s of the slidedecks with video links are shared with participating teachers and parents for their ongoing reference. The slidedecks also contain scripts and implementation tips in the “notes” sections visible to the school clinicians. All intervention groups and meetings occur via remote or hybrid modalities (see Table 1) led by the school clinicians and supported by the trainers who observe in vivo via remote videoconferencing and provide assistance as needed. Trainers also monitor how much of the intervention content school clinicians implement and at what quality level (e.g., clear delivery, effective participant engagement, and appropriate time management). Trainers and school clinicians can interact during the session via the videoconferencing “chat” function; trainers also can provide prompting or modeling by unmuting themselves and speaking aloud. Thus, clinical research team members do not directly serve students, parents or teachers but they may provide support to school clinicians delivering the program during training/consultation meetings and observation of program activities. For a full description of the CLS-R-FUERTE development, training intervention program description, and open-trial findings, see Haack et al. (in press).

### Study Design

We utilized a 2-level (students, schools) cluster RCT design accounting for treatment (CLS-R-FUERTE or SAU) within level 2 (schools); the RCT is registered at clinicaltrials.gov. After baseline data collection activities were complete in each cohort, we randomized schools to CLS-R-FUERTE (school *N* = 4, student *N* = 27) or SAU (school *N* = 4, student *N* = 30) by coding schools with a number to conceal their identity until treatment was assigned and randomizing school numbers to CLS-FUERTE or SAU using a random number generator. Those randomized to CLS-R-FUERTE received the program immediately followed by post assessments for all participants. Subsequently, SAU schools received the CLS-R-FUERTE program in the spring of the 2023 school year. For a detailed description of the CLS-R-FUERTE training and intervention procedures, see Haack et al. (in press).

### Measures

#### Fidelity

CLS-R-FUERTE team members completed fidelity ratings during in vivo remote observation of program activities. Study leads rated CLS-R-FUERTE trainers’ fidelity to the training delivery, and CLS-R-FUERTE trainers supervised by study leads rated school clinicians’ fidelity to the program curriculum based on the amount of content covered (0 = *not at all* to 2 = *fully*; allowing for a calculation of overall percentage of session content covered) and quality (1 = *low* to 5 = *high*, allowing for a calculation of average session quality). This method was successfully used and iteratively updated in previous CLS program trials (Haack et al., 2019, 2020; Pfiffner et al., 2011, 2016, 2023).

##### Attendance

CLS-R-FUERTE team members tracked participant attendance during in vivo remote observation of program activities.

##### Adherence

The primary measure of teacher strategy adherence to the DRC system was the electronic daily Qualtrics survey, which was individualized for each student and prompted teachers to select how many points the student earned on each goal that day. This survey allows for automatic calculation of points which are sent via email to parents, school clinicians, and clinical research team members. Our team then used those survey results to calculate how many days the DRC was used out of the number of possible school days during the program. We also asked parents how often they received DRC point information from teachers on the weekly post-group parent survey (on a 5-point likert scale from “never” to “almost every day.”).

##### Satisfaction

We collected satisfaction ratings by school clinicians, parents, and teachers on post-program questionnaires and calculated how many responded with the two most favorable options on items rated with a 5-point Likert scale (i.e., 4 = satisfied and 5 = very satisfied). We collected student satisfaction via weekly verbal questionnaires (i.e., “how much did you like group today?”) and calculated how many responded with the most favorable option on items rated with a 4-point Likert scale (i.e., 0 = not at all to 3 = a lot).

##### Usability

We collected System Usability Scale (SUS; Brooke, 1996) ratings from school clinicians and parents on weekly and post-program questionnaires. The SUS is a normed 10-item measure with a 5-point Likert scale widely used and adapted for technology usability testing, including evaluations of web-based intervention programs (Lyon et al., 2021) and the remote CLS training program trial in the USA. (Pfiffner et al., 2023). SUS total scores range from 0 to 100, with higher scores indicating better usability and a widely accepted SUS benchmark of good usability at > 68 (Hyzy et al., 2022). The SUS demonstrates strong psychometric properties, including internal consistency (*α* = 0.91) and convergent validity (Bangor et al., 2008).

##### ADHD and ODD Symptoms

Parent and teacher ratings of ADHD and ODD symptom severity were measured using the Child Symptom Inventory, 4th edition (CSI-4; Gadow & Sprafkin, 1997) at baseline and post regardless of treatment assignment. Each ADHD inattentive and hyperactive/impulsive symptom and ODD symptom corresponding to the Diagnostic and Statistical Manual of Mental Disorders (4th ed.; American Psychiatric Association, 2000) is rated on a 4-point scale (0 = never to 3 = very often). Of note, there is not a Spanish version of the Child Symptom Inventory corresponding to the DSM-V or DSM-V-TR; however, the CSI-4 symptoms for school-aged youth reflect the DSM-V-TR criteria (American Psychiatric Association, 2022; Gadow & Sprafkin, 1997). The average severity rating for each diagnosis on the CSI-4 was used in this current study. The English and Spanish versions have demonstrated strong psychometric properties, including test–retest reliability and predictive validity for ADHD and ODD diagnoses (Gadow & Sprafkin, 1997; Sprafkin et al., 2002). The Spanish version was used successfully in the in-person CLS-FUERTE trial in Mexico (Haack et al., 2020) and current study ratings revealed high internal consistency in our sample (*αs* = 0.85 to 0.92).

Given that CLS-R-FUERTE is a school-based program, ADHD and ODD diagnoses were not made and all profile categorization was based on the symptom screening. These procedures are consistent with previous trials of school-based intervention programs (Haack et al., 2019, 2020; Pfiffner et al., 2016, 2023) and supported by research demonstrating a high correspondence between ADHD/ODD symptom screening and clinical diagnoses (Sprafkin et al., 2002). This decision may limit generalizability of current study findings to a clinical population with confirmed ADHD and ODD diagnoses; however, the current study sample likely is reflective of students who would participate in school-based services not requiring formal diagnoses.

##### Functional Impairment

Parent and teacher ratings of functional impairment related to ADHD/ODD (including impairment related to academic and social functioning) were measured using the Impairment Rating Scale (IRS; Fabiano et al., 2006) at baseline and post regardless of treatment assignment. Each impairment item is rated on a seven-point scale (1 = no problem to 7 = extreme impairment) and items were averaged for a total severity score in the current study. The IRS has demonstrated strong psychometric properties including reliability, convergent validity, discriminant validity, and predictive validity for an ADHD diagnosis (Fabiano et al., 2006); the Spanish version was used successfully in the in-person CLS-FUERTE trial in Mexico (Haack et al., 2020) and current study ratings revealed high internal consistency in our sample (*αs* = 0.72 to 0.83).

### Data Analytic Plan

We conducted all data analysis using SPSS version 27, alpha was set at 0.05, and tests were two-tailed unless otherwise noted. We analyzed descriptive statistics (i.e., frequency percentages and mean averages) of program fidelity, attendance, adherence, satisfaction, and usability. The SPSS GENLIN procedure was used to model generalized estimating equations (GEE) using an independent correlation matrix comparing outcomes of CLS-R-FUERTE to SAU while controlling for school clustering. Models were run separately for parent and teacher ratings of three domains at post: ADHD symptoms, ODD symptoms, and overall impairment. Initial models included main effects for baseline ratings and treatment group as well as the interaction effect. Follow-up analyses also included the following covariates: parent level of education, child age, child sex, and ADHD medication status. However, interaction effects were not significant and inclusion of covariates did not alter the pattern or interpretation of results. Therefore, results of models without interaction terms or covariates are presented. The Benjamini–Hochberg correction procedure was used to evaluate significance of the models while controlling for family-wise Type 1 errors given the multiple outcomes examined (Benjamini & Hochberg, 1995). Finally, Hedges’ *g* effect sizes characterizing the between groups effect are presented also presented. Hedge’s *g* is appropriate for small sample sizes; estimates are interpreted consistent with Cohen’s *d* effect sizes (i.e., 0.2 = small, 0.5 = moderate, 0.8 = large).

## Results

As seen in Table 3, CLS-R-FUERTE trainers covered 100% of the initial and weekly training/consultation content at quality levels averaging 4.88 out of 5. School clinicians covered 97% of the parent-group content at quality levels averaging 4.72 out of 5. They covered 98% of the student-group content at quality levels averaging 4.75 out of 5.

Participant attendance averaged 72% for parent groups and 83% for student groups. Given that parents were not typically on-site at the school, they were encouraged to attend group remotely; less than 5% of parents were unable to connect via videoconferencing and chose to attend the groups in-person at their child’s school. In contrast, given that student groups occurred during the school day and students attended school on-site, they were encouraged to attend group in-person; less than 5% of students joined the groups remotely in the event they were absent (for example, due to illness). All students (100%) had a DRC meeting. The teacher-completed electronic DRC surveys documented use at 48% of possible school days. Parent-completed post-group surveys starting at week two documented that 72% of parents reported receiving DRC points from teachers almost every day. Of note, many participants described how teachers sometimes messaged parents cellphone with pictures of the completed paper-and-pencil DRCs each day, but forgot or lacked time for the extra step of electronic DRC survey completion. All school clinicians and parents (100%), as well as 91% of teachers, reported they were “satisfied” or “very satisfied” with the program; all students (100%) reported liking the group “pretty much” or “a lot.” In addition, 100% of school clinicians and 83% of parents reported good CLS-R-FUERTE usability with SUS ratings reaching at least 68 out of 100 over the course of the program.

### Outcomes

All participants in both groups improved significantly on all outcomes over the course of the study. However, students randomly assigned to CLS-R-FUERTE demonstrated greater improvement on parent-rated ADHD symptom severity, ODD symptom severity, and overall impairment as well as teacher-rated ADHD symptom severity (see Table 4). Improvements in teacher-rated ODD symptom severity and overall impairment were not significantly different for students who received CLS-R-FUERTE compared to SAU. A large treatment effect for teacher-rated ADHD symptom improvement and a moderate treatment effect for parent-rated ADHD symptom improvement was observed; all other effect sizes were small.

## Discussion

The current study documents the first known RCT of a comprehensive digitally enhanced school clinician training and ADHD/ODD intervention program in Latin America. Findings supported the feasibility and acceptability of CLS-R-FUERTE, as evidenced by high rates of school clinician fidelity to intervention, participant satisfaction and usability, and program adherence comparable with findings from the in-person CLS-FUERTE program (Haack et al., 2020). Importantly, students receiving the remote program demonstrated significantly more change on the majority of examined outcomes compared to those receiving school services as usual, as evidenced by significant main effects of treatment on parent and teacher-rated ADHD, as well as parent-rated ODD and impairment. Regarding the lack of significant main effects of treatment on teacher-rated ODD and impairment, both SAU and CLS-R-FUERTE groups appeared to show substantial improvement over time. This could be explained by the intensive school services many of the SAU students in the current study were receiving. Indeed, 67% of those in the SAU group were part of the “regular education support services unit,” meaning they qualified to receive resources including family social work, individual or group psychological services, and/or learning or communication tutoring during their waitlist period. It seems plausible that these school services would target behavior and academic impairment, reflected on the teacher-rated ODD and impairment outcomes, but not necessarily teacher-rated ADHD symptom improvement. Supporting this theory, teacher-rated ADHD symptom improvement was significantly greater in the CLS-R-FUERTE group compared to the SAU group and demonstrated a large effect size.

Taken together, current study findings suggest that school clinician training and ADHD/ODD intervention delivery can be successfully facilitated with digital technology, extending prior research supporting in-person treatment delivery programs in conjunction with either remote or in-person clinician training (Haack et al., 2019, 2020; Pfiffner et al., 2011, 2016, 2023) and aligning with calls from the field highlighting the feasibility and benefits of telehealth consultation for providers of youth, and particularly those in rural or under-resourced areas (Fortuna, 2022; McCarty et al., 2015; Mitrani et al., 2021). In Mexico, schools in rural areas were previously unlikely to benefit from EBT training (such as the in-person CLS-FUERTE program, which is the only available EBT training opportunity reported by our stakeholders) due to the inability for university partners to travel for on-site training/consultation and in vivo observation of intervention delivery without substantial time and cost investment. Remote training/consultation modalities (such as the CLS-R-FUERTE program) may promote accessible EBT capacity building in any school with internet connectivity regardless of geographical distance from academic training hubs. The significant CLS-R-FUERTE findings across most outcomes examined alongside prior CLS trials also suggest that comprehensive school-based ADHD/ODD interventions are efficacious regardless of whether the services are delivered in-person or facilitated with digital technology, aligning with previous research supporting the utility of digitally enhanced school clinician training and ADHD/ODD intervention programs (Beidas et al., 2012; Khanna & Kendall, 2015; McCarty et al., 2015; Myers et al., 2015; Pfiffner et al., 2023; Vander Stoep et al., 2017; Xie et al., 2013). These findings are notable, given that emerging reports suggest that telehealth options may reduce geographic and socioeconomic barriers to families engaging in traditional mental health services, such as parents being unable to travel far distances or take time off work to participate in clinic-based services (Fortuna, 2022; Mitrani et al., 2021). Further, parents in CLS-R-FUERTE provided overwhelmingly favorable responses about their experiences, which is similar to emerging reports that youth and parents generally like and are comfortable with telehealth options (McCarty et al., 2015; Mitrani et al., 2021; Myers et al., 2015, 2017; Xie et al., 2013). In summary, the current study and previous research findings converge to support the promise of school-based digitally-enhanced mental health programs for youth with ADHD/ODD and their families.

### Limitations

Several limitations of the current study should be noted and considered in future research. To begin, although we were able to examine current study results alongside prior in-person program trial findings, our RCT design comparing CLS-R-FUERTE to school services as usual (rather than the in-person CLS-FUERTE program) allowed for efficacy testing but did not allow for direct comparison of the in-person versus remote versions of the program. In addition, our outcomes of interest relied on parent and teacher report which may be subject to informant bias. Objective measures, such as direct observations or school disciplinary records, may be beneficial in subsequent studies. Of note, research activities occurred in one Mexican state (i.e., Sinaloa) in-context of a federally-funded clinical trial, which allowed for the provision of needed technological equipment for participating schools (i.e., one laptop, external modem). This may limit the generalizability of our findings to schools with the required resources. That said, parents were not provided with equipment or internet connectivity but were encouraged to attend remote groups using their personal electronic devices and the vast majority attended groups remotely with very few parents attending groups in-person due to limited internet connectivity. Thus, given that three out of the four cohorts successfully held fully remote parent groups (with only one of the four cohorts holding hybrid parent groups), findings may be generalizable to a wide range of families and program activities appear feasible without school-funded equipment or internet connectivity for parents. Importantly, all CLS-R-FUERTE intervention components were delivered by existing school staff (rather than study team members) who were only compensated by our team for completion of questionnaires; principals at participating schools and district leadership needed to agree to support the training/consultation and intervention activities as part of school staff’s salaried workday.

### Implications

Overall, current study results support the promise of digitally enhanced mental health programs for training school clinicians to implement ADHD/ODD interventions worldwide. The high levels of unmet ADHD/ODD need and limited school clinician EBT training in Mexico (Benjet et al., 2009; Borges et al., 2010; Sanchez-Sosa, 2007; Stark et al., 2010) alongside the history of international university collaboration between UCSF and UAS made Sinaloa an ideal setting for initial CLS-R-FUERTE development, implementation, and evaluation efforts. However, lessons learned could be used to inform the translation of findings and deliverables to digital mental health programs for other disorders and/or other global settings with high levels of unmet need. Given the necessity to consider cultural responsivity in technology-enabled mental health services (Eustis et al., 2023), next steps may include examining barriers and solutions related to school climate and culture, community beliefs about mental health conditions and the value of EBTs, and the ability of school districts to provide technology access and dedicated time for staff training/consultation and intervention delivery. This exploration could inform procedures to adapt digital mental programs aligned with school needs and resources while balancing maintenance of program fidelity and outcomes in context of the Behavioral Intervention Technology model framework (Mohr et al., 2014). Scaled-up investigation of such efforts with trials fully-powered to uncover mechanisms of change and identify key program targets appears warranted. Additionally, investigating methods for incorporating remote EBT training, supervision, and consultation into school clinician education curricula may help cultivate a workforce equipped to implement digital ADHD/ODD interventions on a wide scale. Finally, it may be beneficial to advocate for reallocation of existing school resources to evidence-based digital mental health approaches, such as the CLS-R-FUERTE program, on an international policy level to continue enhancing global EBT access and uptake.

## Figures and Tables

**Table 1:** Training and Intervention Components across Collaborative Life Skills (CLS) program versions

Program	CLS	CLS-Spanish	CLS-FUERTE	CLS-Remote	CLS-R-FUERTE
Component	Pfiffner et al., 2016	Haack et al., 2019	Haack et al., 2020	Pfiffner et al., 2023	Current Study
**School Clinician Training:** Initial 6 to 8-hour workshop, weekly consultation, in-vivo observation and fidelity monitoring of each group and meeting supported by intervention manual and materials	Trainers and school clinicians travel between school sites for in-person group training/consultation; trainers travel to school sites to observe and monitor sessions in-person Real time coaching during session and post-session feedback provided in-person Training strategies include didactic instruction and role plays Manuals/materials kept in large paper binders and include paper scripts and handouts of intervention strategies			Training/consultations and sessions observed and monitored *remotely via videoconferencing* Real time coaching during sessions and post-session feedback provided *remotely via video-conferencing* Training strategies adapted for *video-conferencing* target school clinician engagement during remote consultation, *skill demonstration video* review and discussion added to didactic instruction and role play training strategies *Digital* manuals/materials allow 24-hour access from any location and include *electronic scripts and handouts with infographics and video demonstration* of intervention strategies	
**Parent Skill Groups:** Weekly sessions on relationship building, setting expectations, and providing consistent positive and negative consequences	Parents travel to schools for in-person group session delivery				Parents encouraged to attend groups *remotely via video-conferencing* but those unable to connect may attend groups in-person for hybrid group session delivery when needed
**Student Skill Groups:** Weekly sessions on social, emotional, and organizational skills, including good sportsmanship, self-control and cool-down tools, and daily routines	Students must be present at school to attend in-person group sessions Session strategies include discussion and practice of child skills				Students typically attend groups in-person during the school day but those absent from school may attend groups *remotely* for hybrid group session delivery when needed Session strategies include *video demonstration* of child skills
**Teacher Consultation:** Initial 1-hour orientation, initial 20–30 minute meeting to set up a Daily Report Card (DRC), and daily use of the DRC and reporting to parents	Parents travel to schools for in-person DRC meeting delivery Paper-and-pencil DRC travels between school and home each day in the child’s backpack so points can be communicated				Parents can attend DRC meeting *remotely via video-conferencing* Paper-and-pencil DRC is used in the classroom and points are communicated between school and home *electronically*

**Table 2:** Student and parent demographics

	CLS-R-FUERTE (*N*=27)	SAU (*N* = 30)	*p*
**Students (*N* = 57)**			
Female (*N, %)*	4, 15%	11, 37%	.06
Age *(N, %)*^b^			.64
6–7	12, 44%	12, 40%	–
8–9	9, 33%	14, 47%	–
10–12	5, 19%	4, 13%	–
Grade *(N, %)*			.81
1–2	12, 44%	10, 33%	–
3–4	9, 33%	15, 50%	–
5	6, 22%	5, 17%	–
On medication *(%)*	5, 19%	3, 10%	.29
ADHD presentation *(N, %)*^a^			.42
Inattentive profile	6, 22%	5, 17%	–
Combined profile	21, 78%	25, 83%	–
ODD presentation (*N*, %)^a^	12, 44%	16, 53%	.34
**Parents (*N* = 57)**			
Relation to child^b^			.24
Biological mother *(N, %)*	25, 93%	28, 93%	–
Adopted mother	1, 4%		–
Other		2, 7%	–
Marital status *(%)*			.42
Married or cohabitating	24, 89%	14, 47%	–
Widowed	0, 0%	2, 6%	–
Divorced or separated	3, 11%	5, 17%	–
Employment status *(N, %)*			.23
Working full-time	5, 19%	5, 17%	–
Working part-time	2, 7%	6, 20%	–
Stay-at-home parent	14, 52%	8, 27%	–
Unemployed	1, 4&	4, 13%	–
Other	5, 19%	7, 23%	–
Education level *(N, %)*^b^			.35
Less than high school	8, 30%	13, 43%	–
High school degree	8, 30%	3, 10%	–
Some college	3, 11%	5, 17%	–
College degree	4, 15%	6, 20%	–
Advanced degree	3, 11%	2, 7%	–

All participants represented White race, Latino/a ethnicity

*ADHD* Attention-Deficit/Hyperactivity Disorder, *ODD* Oppositional Defiant Disorder

a ADHD and ODD symptoms presentation profiles based on number of symptoms endorsed by parents OR teachers on the CSI-4 (Gadow & Sprafkin, 1997) at baseline

b Indicates some missing data due to participants choosing not to report on that demographic factor. CLS-R-FUERTE = treatment group SAU = School services as usual

**Table 3:** Fidelity, attendance, and adherence for CLS-R-FUERTE treatment condition (SAU excluded)

Measurement	Rater	CLS-R-FUERTE (N = 27)	CLS-FUERTE (N = 28)
CLS-R-FUERTE Trainer Fidelity	Study Team Lead observer	100%, high competence (4.88 of 5)	
School Clinician Attendance	Objective Frequency	100%	
**Parent group component**			
Provider Fidelity	CLS-R-FUERTE Trainer Observer	97%, high competence (4.72 of 5)	97%, high competence (4.53 of 5)
Participant Attendance	Objective Frequency	72% (range = 14–100%)	76% (range = 0–100%)
Satisfaction	Parent	100% “satisfied” or “very satisfied”	98% “satisfied” or “very satisfied”
**Student group component**			
Provider Fidelity	CLS-R-FUERTE Trainer Observer	98%, high competence (4.75 of 5)	93%, high competence (4.36 of 5)
Participant Attendance	Objective Frequency	89% (range = 57–100%)	94% (range = 83–100%)
Satisfaction	Student	100% liked the group “pretty much” or “a lot”	93% liked the group “pretty much” or “a lot”
**Classroom component**			
DRC Use	Electronic Tracking	2.4 out of 5 days	4.3 of 5 days
DRC Use	Parent	72% report receiving points “most days”	
Parent-Child-Teacher DRC Meeting	Objective Frequency	100% at least 1	100% at least 1
Satisfaction	Teacher	91% “satisfied” or “very satisfied”	98% “satisfied” or “very satisfied”

*CLS-R-FUERTE* remote program of interest in current study, *CLS-FUERTE* in-person program (Haack et al., 2020), *SAU* School services as usual

**Table 4:** Descriptives and treatment outcomes controlling for school cluster

Outcome	CLS—R-FUERTE (*N* = 27)	SAU *(N*=*30)*	Main effect baseline	Main effect group	Between groups ES^d^
	*M (SD)*	*M (SD)*	*B (SE)*	*B (SE)*	*g*
*Parent-rated ADHD symptom severity*^ab^			0.52 (0.10)^c^	0.35 (0.10)^c^	
Baseline	1.73(0.63)	1.90(0.60)			0.48
Post-treatment	0.96 (0.51	1.43(0.60)			
*Teacher-rated ADHD symptom severity*^ab^			0.62 (0.11)^c^	0.55 (0.17)^c^	
Baseline	1.91(0.63)	1.81 (0.59)			0.97
Post-treatment	1.20 (0.74)	1.69(0.60)			
*Parent-rated ODD symptom severity*^a^			0.32 (0.12)^c^	0.34 (0.09)^c^	
Baseline	0.99 (0.63)	1.31 (0.68)			0.19
Post-treatment	0.67 (0.47)	1.10 (0.53)			
*Teacher-rated ODD symptom severity*^b^			0.75 (0.15)^c^	0.08 (0.25)	
Baseline	1.19(0.69)	1.20 (0.90)			0.11
Post-treatment	0.88(0.77)	0.89(0.86)			
*Parent-rated Impairment* ^%ab^			0.23 (0.07)^c^	0.62 (0.17)^c^	
Baseline	4.03(1.59)	4.03(1.33)			0.41
Post-treatment	1.72(0.80)	2.36(0.98)			
*Teacher-rated impairment*^ab^			0.59 (0.19)^c^	0.59 (0.42)	
Baseline	5.01 (1.22)	5.18(1.08)			0.37
Post-treatment	2.90 (1.51)	3.59 (1.46)			

*ADHD* Attention Deficit/Hyperactivity Disorder, *ODD* Oppositional Defiant Disorder, all analysis by original assigned groups, *CLS-R-FUERTE* treatment group, *SAU* school services -as-usual group, *ES* effect size

a Significant in original CLS trial

b Significant in CLS-FUERTE trial

c Significant after within-domain Benjamini–Hochberg false discovery rate correction

d Standardized mean differences corrected for sample size bias (Hedges’ *g*)
